# The potential for mitigation of methane emissions in ruminants through the application of metagenomics, metabolomics, and other -OMICS technologies

**DOI:** 10.1093/jas/skab193

**Published:** 2021-09-29

**Authors:** Victoria Asselstine, Stephanie Lam, Filippo Miglior, Luiz F Brito, Hannah Sweett, Leluo Guan, Sinead M Waters, Graham Plastow, Angela Cánovas

**Affiliations:** 1 Centre for Genetic Improvement of Livestock (CGIL), Department of Animal Biosciences, University of Guelph, Guelph, Ontario, N1G 2W1, Canada; 2 Department of Animal Sciences, Purdue University, West Lafayette, IN, 47907, USA; 3 Livestock Gentec, Department of Agricultural, Food and Nutritional Sciences, University of Alberta, Edmonton, Alberta, T6G 2C8, Canada; 4 Animal and Bioscience Research Department, Teagasc Grange, Dunsany, Co. Meath, C15 PW93, Ireland

**Keywords:** genomic selection, metabolomics, metagenomics, methane emissions, ruminants

## Abstract

Ruminant supply chains contribute 5.7 gigatons of CO_2-eq_ per annum, which represents approximately 80% of the livestock sector emissions. One of the largest sources of emission in the ruminant sector is methane (CH_4_), accounting for approximately 40% of the sectors total emissions. With climate change being a growing concern, emphasis is being put on reducing greenhouse gas emissions, including those from ruminant production. Various genetic and environmental factors influence cattle CH_4_ production, such as breed, genetic makeup, diet, management practices, and physiological status of the host. The influence of genetic variability on CH_4_ yield in ruminants indicates that genomic selection for reduced CH_4_ emissions is possible. Although the microbiology of CH_4_ production has been studied, further research is needed to identify key differences in the host and microbiome genomes and how they interact with one another. The advancement of “-omics” technologies, such as metabolomics and metagenomics, may provide valuable information in this regard. Improved understanding of genetic mechanisms associated with CH_4_ production and the interaction between the microbiome profile and host genetics will increase the rate of genetic progress for reduced CH_4_ emissions. Through a systems biology approach, various “-omics” technologies can be combined to unravel genomic regions and genetic markers associated with CH_4_ production, which can then be used in selective breeding programs. This comprehensive review discusses current challenges in applying genomic selection for reduced CH_4_ emissions, and the potential for “-omics” technologies, especially metabolomics and metagenomics, to minimize such challenges. The integration and evaluation of different levels of biological information using a systems biology approach is also discussed, which can assist in understanding the underlying genetic mechanisms and biology of CH_4_ production traits in ruminants and aid in reducing agriculture’s overall environmental footprint.

## Using “-Omics” Technologies to Mitigate Methane Emissions

Ruminants can utilize cellulolytic and hemicellulolytic feedstuffs, which are broken down via prokaryotic and eukaryotic microorganisms (Newbold and Ramos-Morales, 2020). During the breakdown process, hydrogen, carbon dioxide (CO_2_), and formic acid are produced as end products of fermentation (Hook et al., 2010; Danielsson et al., 2017). Within the rumen, the methanogenic archaea use H_2_ to reduce CO_2_ to produce methane (CH_4_), leading to increasing levels of greenhouse gas (**GHG**) (Danielsson et al., 2017). Global demands and initiatives to reduce GHG emissions are being implemented, such as the UK Climate Change Act (Gill et al., 2010; Yan et al., 2010; Sejian et al., 2011), Ontario’s (Canada) GHG initiatives to reduce GHGs based on the 2007 climate change action plan (Ontario, Go Green, 2007) which has been successfully completed and the Intergovernmental Panel on Climate Change (**IPCC**) Paris agreement on climate change which aims to limit the climate warming to 2 °C (Nisbet et al., 2019). Of the major GHGs, CH_4_, which is a product of enteric fermentation from ruminants, is regulated by multiple physiological and environmental factors (Manzanilla-Pech et al., 2016). Approximately 40% of agricultural emissions in Canada come directly from CH_4_, with ruminants contributing up to 90% of this CH_4_ production (Agriculture and Agri-Food Canada, 2019). Various CH_4_ mitigation strategies are being investigated to lower CH_4_ levels produced by ruminants, such as the manipulation of dietary formulations (Caro et al., 2016) and chemical inhibitors to reduce rumen methanogenesis (Kumar et al., 2014). Despite the effectiveness of such approaches, the use of genetic and genomic selection to breed for reduced CH_4_ production is expected to provide substantial genetic gains, which are cumulative and permanent over generations (de Haas et al., 2011; González-Recio et al., 2020). Breeding for lowered CH_4_ emissions has been shown to be an effective approach in sheep (Rowe et al., 2019) Low CH_4_ sheep in a 10-yr study in New Zealand grew more wool, had smaller rumens with different microbiomes, were leaner, and had a different fatty acid profile in the muscle, which are more economically sustainable compared to high CH_4_ sheep (Rowe et al., 2019).

To achieve genetic progress for complex traits, the genetic mechanisms and underlying biology must be well understood. The recent availability of “-omics” technologies at lower costs has contributed to the generation of larger datasets, which are being used to improve the understanding of the underlying functional biology of economically relevant traits such as feed efficiency (**FE**) and its association with CH_4_ in ruminant species (Herd et al., 2016; Løvendahl et al., 2018; Seymour et al., 2019; Sun et al., 2019). These “-omics” techniques include metagenomics, which allows for the evaluation of taxonomic and genetic composition, and functionality of microbial communities, and has been used to help elucidating the relationship between host phenotypes and microbial diversity and density in the rumen (Morgavi et al., 2013; Denman and McSweeney, 2015; Malmuthuge and Guan, 2016, 2017). In addition, metabolomics is a multidisciplinary approach integrating biostatistics, biochemistry, and bioinformatics, and allows for metabolite profiling that can help identify biomarkers reflecting physiology. For example, metabolomics has been used to highlight biological processes regulating milk production and other economically relevant traits (Fontanesi, 2016). Metagenomics and metabolomics coupled with other “-omics” data sources such as genomics, transcriptomics, and proteomics, in an integrated systems biology approach are critical to understanding the biological architecture of complex traits and molecular modifications in response to internal and external factors (Deusch et al., 2015; Sun et al., 2020). The integration of complementary data sources may lead to improved genetic progress for complex traits and contribute to the long-term economic benefits and sustainability of ruminant production systems (Denman and McSweeney, 2015; Sun et al., 2020).

Using approaches that integrate -omics data is especially beneficial when studying complex traits, which are influenced by multiple genes and genetic features and therefore can be better understood when studying the trait as a biological system. Such approach can determine relationships between phenotypic, genetic, and metabolic features, and reveal causal regulatory mechanisms. Systems biology approaches have taken top-down and bottom-up strategies to understand successive levels of biological information and how it influences a system (Shahzad and Loor, 2012). As CH_4_ is a product of rumen fermentation and methanogenic archaea, the approach to identify animals with a genetic profile or select for specific pathways or species within the microbiome to reduce CH_4_ production would apply a top-down approach. Reconstruction of this system using a top-down approach involves sampling of host tissues, as well as rumen fluid, contents, and tissue, use of high-throughput sequencing (i.e., genomics, metagenomics, transcriptomics, metatranscriptomics, metabolomics), statistical analysis, and biological interpretation (Shahzad and Loor, 2012). Various statistical models have been applied such as Bayesian gene networks, following a top-down approach by associating genotypes and operational taxonomic units (**OTU**) profiles (Parviainen and Kaski, 2017). By using a top-down approach, we can continue to use host genetic information and associate this level with rumen microbial community structure and function. Ways to implement multi-omics top-down approach can involve the collection of information about genes, genetic variants, and metabolites of the host and rumen microbiome.

Analyses used to integrate -omics data at different levels to determine associations include co-expression matrix analysis, such as causal Bayesian gene networks (Parviainen and Kaski 2017), and co-expression modules (i.e., clusters) that identify eQTLs for different expression data types (genotype, gene, protein, and/or metabolite expression data) (Kadarmideen, 2016). More specifically, to study causal regulatory mechanisms, Bayesian networks are used to understand conditional dependencies and independencies between random variables by modeling gene regulation networks using statistical dependency models (Parviainen and Kaski, 2017). These Bayesian networks are capable of modeling multiple different measurements of expression of the same genes, without being biased by data originating from different platforms. Recently, Bayesian modeling has been used to assess associations between host genetics and rumen microbiota and its influence on CH_4_ emissions in dairy cattle (Zhang et al., 2020), using genotypes and OTU classification. This study revealed that the host genetics genome and rumen microbiota explain 25% and 7% of CH_4_ emissions, respectively. Additionally, specific host genes were significantly associated with rumen microbiota composition (Zhang et al., 2020). This provides evidence that applying integrated approaches to assess associations between host genetics and rumen microbiome can provide insights on their collective influence on CH_4_ emission variation. Other multi-omics approaches, termed as “systems genetics” have combined genomics and transcriptomics to model biological networks and reconstruct causal gene networks using markers identified from genome-wide association study (**GWAS**; Civelek and Lusis, 2014). These studies suggest the emerging computational and statistical analyses being applied to evaluate relationships between the genome, transcriptome, and metabolome of the host and rumen microbes. A complete understanding of several aspects of genomic, metabolomics and metagenomics research, as well as the current understanding of CH_4_ emissions research using the latter tools is needed for studying and applying of integrative -omics approaches. This comprehensive review discusses the methods for measuring CH_4_, and the generation, analytics, and value of integrating genomics, metabolomics, and metagenomics information to mitigate the effects of climate change by reducing GHG emissions from ruminants.

## Measuring Methane Emissions in Ruminants

There are numerous ways to assess CH_4_ emission in ruminants, for example, using direct measurements, proxies, or prediction equations (Herd et al., 2014; Negussie et al., 2017; Huws et al., 2018). One method is to measure the tracer gas sulfa hexafluoride (SF_6_) which is nontoxic, physiologically inert, and stable (Lester and Greenberg, 1950; Johnson et al., 1992). Similar to CH_4_, SF_6_ is capable of mixing with rumen air, and is inexpensive to detect and analyze. However, the main issue with SF_6_ is the use of permeation tubes, which require precise calculation of the release rate of SF_6_. The accuracy of release rate calculation is critical for CH_4_ determination and tubes with high release rates represent higher CH_4_ emission recordings, which leads to a high error rate (Storm et al., 2012). Comparably, a more extensive but more accurate method to measure CH_4_ includes respiration chambers, which have been used for the past century to study metabolic energy use in animals (Mclean and Tobin, 1987; Johnson et al., 2003). Respiration chambers are considered the gold standard methodology for measuring CH_4_ from individual animals as they enable accurate and repeatable CH_4_ measurements in ruminants (Leahy et al., 2013; Jonker et al., 2018). However, this method is costly, complex, labor intensive, and has a low capacity for number of animals that can be measured at one time, thus limiting the availability for large-scale application. Furthermore, it does not reflect the living conditions of the animals in production systems (Storm et al., 2012). Another method is the GreenFeed machine (C-Lock Inc, 2015), which is an automatic feeding system and CH_4_ analyzer that measures daily CH_4_ and CO_2_ mass fluxes from the breath and eructation gas of individual cattle for ranking them based on their daily measurements (Zimmerman et al., 2011; Hristov et al., 2015). Automatic feeding systems allow ear tags or collars to be read and measurements for all cows to be recorded while the animal is consuming feed. This method can be applied in conventional tie-stalls, and for grazing animals fed supplements in feeders. A limitation with this system is that it is only able to measure CH_4_ when the animal’s head is in the feeder (Storm et al., 2012). Another technique that measures CH_4_ includes micrometeorological techniques. One such method is the combination of long line-averaging concentration sensors (i.e., open path lasers) and inversion-dispersion methods which allow animals to be measured in their natural environment (Flesch et al., 2019). One reported issue with this method is the need to know the location of the emission source(s), i.e., the animal, however this issue has been limited by narrowing the paddock. Using a narrow paddock also increases the range of useable wind directions, thus increasing the likelihood of a measurable concentration downwind of the paddock (Flesch et al., 2019). Another method to determine CH_4_ is through proxies, such as feed intake, milk production and composition, and feces. A review by Negussie et al. (2017) provides a detailed assessment of proxies for measuring CH_4_, therefore, this section provides a general overview on major techniques used for CH_4_ prediction. There is increased interest in using proxies as indicators of CH_4_, including measures of milk fatty acids, milk mid-infrared (**MIR**) spectroscopy, and rumen metabolites (i.e., Dehareng et al., 2012; Vanlierde et al., 2015; Negussie et al., 2017). In general, practical proxies need to be accurate, inexpensive, and able to record on a large-scale basis (Negussie et al., 2017). The combination of proxies is an efficient and accurate way to predict CH_4_; however, the combination of robust and applicable proxies across a variety of environments is challenging. Lastly, prediction equations can also be used to predict CH_4_. A primary prediction used for CH_4_ measurement proposed by Blaxter and Clapperton (1965) used gross energy intake, apparent digestibility of dietary energy at maintenance (%), and level of energy intake, relative to that required for maintenance. However, this prediction equation is no longer used due to substantial changes in the genetic merit of the animals, which would require new validation studies. There are also other numerous prediction equations available, and the most accurate ones included dry matter intake, metabolizable energy intake, acid detergent fiber intake, and lignin intake (Sobrinho et al., 2019).

In summary, there are multiple methods to measure CH_4_, however, each method has its associated advantages and limitations. Therefore, careful consideration is required before selecting an optimal method to use in future studies based on experimental design and strategies. Similarly, when looking at CH_4_ emissions in individual animals, it is important to consider not only the amount of CH_4_ produced, but the amount of feed consumed and milk/meat produced, as this can be indicative of animal efficiency.

## Genomic Selection for Reduced Methane Emissions in Ruminants

Observed phenotypes are the result from the host genetic-makeup and environmental factors (i.e., FE or CH_4_). Methane production in ruminants is heritable with estimates ranging from 0.12 to 0.52 (de Haas et al., 2011; Kandel et al., 2012; Pickering et al., 2015a; Brito et al., 2018), indicating that genetic progress can be made through selective breeding. Reducing CH_4_ by improving genetic merit of the herd is a cost-effective strategy to decrease CH_4_, although, under practical conditions, measuring CH_4_ remains challenging (de Haas et al., 2011, 2017). Genomic selection has been implemented to select for difficult-to-measure traits such as hoof lesions in Holstein cattle (Dhakal et al., 2015), fertility in Holstein cattle (Guarini et al., 2019), and mastitis in meat sheep (McLaren et al., 2018) and thus could be implemented for reduced CH_4_ (Hayes et al., 2013). However genomic selection requires a sizeable training population (i.e., animals that have both genotypic data and measurements for CH_4_) to be successfully implemented. Additionally, limitations should be considered, such as training populations for specific livestock that commonly have crossbred or mixed populations (i.e., sheep and beef cattle), which may result in less accurate and more biased genomic predictions unless larger and well-structures training populations are designed, and breed composition is accounted for. Another factor to consider is antagonistic effects between CH_4_ emissions and desirable production traits, which should be considered when selecting for CH_4_ reduction (Breider et al., 2019; Pszczola et al., 2019; González-Recio et al., 2020).

The relatively small number of CH_4_ records in genotyped animals has limited investigation of this phenotype (de Haas et al., 2011, 2017; Hayes et al., 2013, 2016; Rowe et al., 2014). Furthermore, defining phenotypes and biological indicators of CH_4_ is a major challenge as it should be independent of other physiological and nutritional factors (Rowe et al., 2014), and be easily measured and ideally obtained at a low cost to breeders. A positive and moderate genetic correlation (0.72) between residual feed intake (**RFI**; a common measure of FE) and predicted CH_4_ emissions (**PME**) has been reported (de Haas et al., 2011), suggesting the importance of studying FE in coordination with CH_4_ traits. With the heritability of RFI ranging from low to moderate in beef (0.07 to 0.62; Berry and Crowley, 2013) and dairy (0.04 to 0.36; Jensen et al., 1995; Hurley et al., 2016) cattle, there is potential for genetic improvement in RFI in coordination with CH_4_ (Berry and Crowley, 2013). The use of proxy variables for FE that have moderate to high heritability (de Haas et al., 2011; Pickering et al., 2015a) and for PME with heritability between 0.05 ± 0.03 (Pickering et al., 2015b) and 0.35 ± 0.12 (de Haas et al., 2011), may be of great value to increase the rate of genetic progress for reduced CH_4_ (Verbyla et al., 2010; de Haas et al., 2011). To enlarge datasets for direct selection for reduced CH_4_, an international effort is necessary to jointly gather feed intake and CH_4_ records in ruminants. International projects and initiatives currently exist that promote use of accessible data across countries in order to reach a sufficiently sized training population; such projects and initiatives include METHAGENE (www.methagene.eu), the Efficient Dairy Genome Project (http://genomedairy.ualberta.ca/), and the Resilient Dairy Genome Project (http://www.resilientdairy.ca).

Genomic selection can be enhanced which a systems biology approach incorporating both metagenomics and metabolomics, to identify host functional genes, biomarkers, and rumen microbial genes potentially associated with CH_4_ to be identified. A complementary approach to identify important genomic regions and candidate genes associated with traits of interest is known as GWAS. For instance, a GWAS performed for CH_4_ yield and gross CH_4_ in sheep identified two significant markers harboring the candidate genes *Tetraspanin 14* (***TSPAN14)*** which has been significantly associated with inflammatory bowel disease in human (Jostins et al., 2013) and *Peroxisomal Biogenesis Factor 2 (****PEX2***) which is hypothesized to be important to lipid metabolism and fatty acid oxidation in cattle (Mach, 2013; Rowe et al., 2014). Thus, Rowe et al. (2014) concluded that due to rumen fermentation involving fatty acid metabolism and the complex microbial community of the human colon, both are good physiological candidates for CH_4_ yield. Therefore, using systems biology and GWAS, information can be incorporated in genomic prediction approaches to improve selection strategies aimed to reduce CH_4_ (Saleem et al., 2013; Ursell et al., 2014; Cánovas, 2016). As mentioned previously, high selection pressure on specific traits should be considered due to antagonistic effects on other commercially important traits. However, this emphasizes the importance to consider approaches that help to better understand CH_4_ emissions across multiple -omics levels. Furthermore, various environmental factors must be considered such as nutritional and management practices (Manzanilla-Pech et al., 2016).

## Metagenomics and Metabolomics as Additional Approaches to Mitigate Methane Emissions in Ruminants

The use of metagenomic and metabolomic approaches to understand the variation in microbial genomes and microbial profiles has provided important insights to the contribution of rumen microbial structure and metabolite profiles (Attwood et al., 2008; Goldansaz et al., 2017); as well as the regulation of complex phenotypes such as FE, CH_4_, and nitrogen retention (Bath et al., 2013; Artegoitia et al., 2017; Meale et al., 2017). Metagenomics provides information on relative abundance and identification of microbial species and genes, which has advanced the evaluation of taxonomic and genetic composition of complex microbial communities. This has enabled the identification and quantification of microbial data to describe microbial community structure and functions. Microbiomes consist of many genomes compared to the host, and the microbial structure of microbiomes can be studied using metagenomics (Te Pas et al., 2017). In ruminant research specifically, metagenomics has been used to determine sequence information from microbes to elucidate the relationship between microbial diversity and density with host phenotypes in cattle and sheep (Shi et al., 2014; Denman and McSweeney, 2015; Wallace et al., 2015; Huws et al., 2018; Saborío-Montero et al., 2019). However, the use of metagenomics to define microbiomes when studying potential associations between host microbial profiles with CH_4_ was first carried out in cattle in 2018 (Difford et al., 2018). This study revealed that variation in CH_4_ is influenced 21% by the host genome and 13% by microbiota composition. It was also found that the host genome and rumen microbes were largely independent from each other, which suggests that breeding for lower CH_4_ emitting animals is unlikely to result in unfavorable changes in the rumen microbes (Difford et al., 2018). A recent study used structural equation models to integrate genomic, metagenomic, and phenotypic information to identify the biological relationships between the host–metagenome–phenotype relationships in dairy cattle (Saborío-Montero et al., 2019). They found that positive genetic correlations were estimated consistently for the different phyla including Ciliophora phylym, Euryarchaeota (*Methanobrevibacter sp*.), Chytridiomycota (*Neocallimastix sp.*), and Fibrobacteres (*Fibrobacter sp*.) phyla. Presently, metagenomic sequencing of host microbiomes and microbial genomic sequencing of various sample types, including the rumen microbiome, is expanding (Kim et al., 2017; Stewart et al., 2018; Martínez-Álvaro et al., 2020). The use of metagenomics has allowed for near-complete microbial genomes to be assembled and results in the identification of many sequenced rumen microbial strains and species (Stewart et al., 2018). Additionally, networks such as The Rumen Microbial Genomics Network (www.rmgnetwork.org/), and the development of the reference microbial genome catalog is expanding (Hungate1000 Project, Seshadri et al., 2018). There is also the Global Rumen Census in which 35 countries were involved (Henderson et al., 2015). The Global Rumen Consensus aimed to identify the foregut microbial community in 742 samples, from 32 rumen and camelid species. They found that similar bacteria (*Prevotella*, *Butyrivibrio*, and *Ruminococcus*, as well as unclassified *Lachnospiraceae*, *Ruminococcaceae*, *Bacteroidales*, and *Clostridiales*) and archaea (*Methanobrevibacter gottschalkii*, *Methanobrevibacter ruminantium Methanosphaera sp*. and two *Methanomassiliicoccaceae* group 10 and group 12) dominated in nearly all samples. Thus, making it possible to mitigate CH_4_ emissions by developing strategies to target the few dominant methanogens including vaccines or small-molecule inhibitors (Henderson et al., 2015). Other research by Seshadri et al. (2018) identified and sequenced 410 cultured bacteria and archaea species, increasing the coverage of rumen microbes inhabiting archaeal and bacterial domains. However, there remains a lack of sequence and culture information on specific rumen microbes for reference databases. Studies have used metagenomic and metatranscriptomic approaches at the rumen microbiome level to study CH_4_. For instance, Xue et al. (2020) found that the rumen microbiome of high milk protein cows also had lower relative abundances of organisms with methanogen and methanogenesis functions, which suggest that these animals produce less CH_4_. Martínez-Álvaro et al. (2020) investigated functional niches in the rumen microbiome that affects variation in CH_4_ in high and low emitters. They found that CH_4_ emissions was mainly explained by variables involved in organic matter degradation pathways, rather than only being methanogen driven. Therefore, to improve the accuracy of identifying functional information about the microbial genome, and further interpret their interactions using metagenomics, the genetic diversity, annotation, and accuracy of the microbial reference genomes is necessary. Thus, the incorporation of metagenomic data with functional analysis such as metatranscriptomics and network-based approaches provide integrative information on microbial function and activity (Wang et al., 2017; Li et al., 2017, 2018, 2019).

Metabolomics enables the identification of a network of biological indicators that reflect physiological and pathological events occurring, highlighting phenotypic differences observed between extreme groups of animals. The use of metabolomics in ruminant research has advanced the evaluation of system-wide metabolism and biology (Goldansaz et al., 2017), contributing to the definition of numerous organic molecules present in a biological tissue or fluid. Such metabolites are produced by different biochemical pathways and various enzymatic reactions (Oliver et al., 1998). Additionally, metabolomics highlights biological processes involved in regulating the animal body (Rohart et al., 2012). Through metabolomics, numerous metabolites including milk fatty acid metabolites, volatile metabolites, and nonvolatile metabolites have been identified and metabolomic profiles between animal groups segregated by extreme phenotypes have been compared (Deusch et al., 2015; van Gastelen et al., 2018).

Metabolomic analysis in dairy cattle has largely focused on identifying milk metabolites (Kühn et al., 2014), which can be used to potentially predict CH_4_ production (van Gastelen et al., 2018). van Gastelen et al. (2018) combined all the data collected into four sets of test variables to develop 11 CH_4_ prediction models. They concluded that, of the models they tested, MFA have a moderate potential to predict CH_4_ emission in dairy cattle fed forage-based diets, while it is not worthwhile to use volatile and nonvolatile metabolites to estimate CH_4_ emission. In general, metabolites from rumen fluid, blood, and blood plasma show a wide range of variation due to genetics and physiological status (Suhre and Gieger, 2012; Artegoitia et al., 2017; Meale et al., 2017). In relation to CH_4_ production, rumen fluid is critical as it contains fermentation end products for host utilization, contributing directly to GHG emissions (Moss, 1993; Newbold et al., 1995). Wang et al. (2018) reported that rumen fluid from slaughtered animals may be a useful method to study variations in CH_4_ from different cattle types (dairy vs. beef) and ages. Additionally, the relationship between the rumen microbiome and the host phenotype have been better understood by characterizing the metabolomic profiling from divergent groups relating to disease (Adamski and Suhre, 2013), FE (Artegoitia et al., 2017), diet supplementation or transition (Mao et al., 2014; Golder et al., 2018), and milk protein yield (Xue et al., 2020). Guan et al. (2008) compared two divergent groups, high- and low-RFI animals, and found the total volatile fatty acid concentration in low-RFI animals to be almost doubled (*P* = 0.059) in rumen fluid compared to high-RFI steers. Thus, indicating more active microbial fermentation and relatively higher CH_4_ production in low-RFI steers. Due to the differences observed between groups, rumen fluid serves as a valuable method to collect information on metabolites and their association with CH_4_ production (Guan et al., 2008). However, the integration of metabolomic data from several sample types is important for interpreting biological networks and metabolic pathways (Goldansaz et al., 2017). Thus, the characterization of livestock metabolites is currently expanding and incorporated into the metabolite database for livestock research (Livestock Metabolome Database [**LMDB**]; www.lmdb.ca).

## Metagenomics

### Platforms and technologies available for metagenomics

Advances in molecular genetic techniques using next-generation sequencing (**NGS**) technology, has enabled the study of culture independent and complex microbial communities (Delgado et al., 2019), due to its high-throughput DNA sequencing methods using massive parallel sequencing of millions of DNA molecules. NGS has allowed for different yields and sequence lengths to be identified after a single run at a high-throughput level (Escobar-Zepeda et al., 2015). It requires high read depth, but also allows for unbiased microbial sequencing (Sharpton, 2014). Additionally, it can perform untargeted massively parallel sequencing of microbes present in biofluids, generating quantitative microbial profiles, for example for rumen microbiomes (Ross et al., 2013). An approach within NGS technology includes metagenomic sequencing, which has been used to study rumen microbiomes. The identification, quantification, and functional annotation of metagenomic sequences resulting in the discovery of a vast amount of microbial diversity have been possible due to metagenomic sequencing. Currently, multiple platforms and technologies are available for metagenomic sequencing of rumen microbial genomes. Major metagenomic platforms are summarized in Table 1.

**Table 1.** Methods used and/or currently used to measure metagenomes, and significant benefits and limitations for metagenomic analysis

Technique	Benefit	Limitation
Traditional DNA Sequencing	Provide long continuous reads Targeted and specific	Laborious and time consuming Amplification bias Inefficient
Amplicon Sequencing	Characterize target sequences (i.e., 16s rRNA, 18s rRNA genes, OTUs)	Low-throughput method
Shotgun Sequencing	High-throughput Massive parallel sequencing Unbiased microbiome profiling	Require high sequencing depth
RNA Sequencing	High sensitivity and reproducibility Require low sequencing depth	Require biological replicates Accuracy depends on annotation quality of reference genome

Metagenomic analyses can identify the microbes (species, phylum identification, and abundance) present in the rumen and their activity (i.e., through functional annotation for genes and pathways). In addition, modeling of kinetics and dynamics of the rumen microbiome should also be explored to understand and appropriately assess the function of the rumen microbiome and its association with other -omics levels. More recently, the Illumina MiSeq system (Illumina Inc., San Diego, CA, USA) has been used for metaxonomic sequencing of small genomes such as bacteria, while reducing error rates by up to two magnitudes (Kozich et al., 2013; Reuter et al., 2015). Application of the Illumina MiSeq platform is also commonly used for 16S rRNA gene sequencing of rumen microbiota (Acinas et al., 2004). Metagenomic analysis has also been applied using shotgun sequencing, which allows for the fragmentation and sequencing of the entire genome of an organism (Sharpton, 2014).

These high-throughput analyses have advanced traditional polymerase chain reaction (**PCR**) and RNA/DNA amplicon-based sequencing (amplicon-seq) metagenomics, by providing more efficiency and throughput. Traditional quantitative polymerase chain reaction (**qPCR**) is known to be efficient, inexpensive, and specific, for target analysis, but has low-throughput capability and is therefore not as beneficial for metagenomic analysis. Comparably, RNA/DNA amplicon-seq, which is now considered a metataxonomic analysis, of phylogenetic marker genes has been used for profiling microbial communities using 16S rRNA/rDNA gene amplicon data for prokaryotic organisms (bacterial and archaeal sequencing) or 18S rRNA/rDNA gene amplicon data and Internal Transcribed Spacer (**ITS**) region amplicon for eukaryotic organisms or rumen fungi (Giovannoni et al., 1990; Escobar-Zepeda et al., 2015; Fuyong et al., 2016). Specifically, amplicon-seq for fungi sequencing includes sequencing of ITS region; as the 18S rRNA gene contains more hyper variable regions, ITS sequencing allows for maximized coverage of hyper variable ITS domains which is more suitable for identifying fungi (Bokulich and Mills, 2013). In contrast, 16S rRNA gene amplicon data allow for the characterization of OTUs, which represent clusters of similar bacterial and archaeal sequences and are used to classify bacteria and archaea. Operational taxonomic units profiles and abundance have been useful in characterizing bacterial profiles across cattle divergent for FE (Carberry et al., 2014a, b; McGovern et al., 2018; Paz et al., 2018). However, this sequencing method has limited resolution and low sensitivity (Poretsky et al., 2014). Overall, amplicon-seq has allowed for the characterization of 16S rRNA and 18S rRNA genes, providing more information on microbial diversity. Use of this technology has identified microbial and rumen fermentation markers in the methanogenic community associated with divergent RFI groups (McGovern et al., 2020). However, to generate a deeper characterization, shotgun metagenomic analysis is more suitable for metagenomic analysis, as it can lead to improved classification of OTUs compared to other approaches such as amplicon-seq (Hao et al., 2012; Hilton et al., 2016). Additionally, amplicon-seq for 16S rRNA phylogenic profiling of rumen microbiome bacteria and archaea must consider multiple factors that could limit accuracy and specificity in results, such as the DNA extraction method used, choice of hypervariable regions of marker genes, PCR conditions and so on (McGovern et al., 2018).

Metagenomic-assembled genomes (**MAGs**) can also be used to assess trait associations with compositional and functional potential of the rumen microbiome. Archaeal genomes of the ruminant rumen microbiome exist, with methanogenic archaeon classified. However, more research is needed to better understand the relationship between methanogens and CH_4_ emissions, as proportional differences in methanogenic archaeon’s does not directly associate with CH_4_ production (Stewart et al., 2019).

In addition to metagenomic sequencing and MAGs, high-throughput RNA-Sequencing (**RNA-Seq**) has recently been used for profiling of RNA molecules isolated from microbiome (Cottier et al., 2018), and is termed as metatranscriptomics in studies of transcriptomics of microbes. Metatranscriptomics is based on mRNA quantification using RNA-Seq and can measure differentially expressed genes by sequencing mRNA and mapping to a reference sequence (Mortazavi et al., 2008; Pightling et al., 2015). It can identify both the microbial species present in a sample as well as providing information for gene expression and functional annotation of rumen microbes. Recently, meta-total RNA-Seq has been proven to provide the highest sensitivity and accuracy for bacteria and fungi profiling compared to other methods (Cottier et al., 2018). In addition, RNA-Seq requires lower sequencing depth compared to shotgun metagenomics (Cottier et al., 2018). Overall, the use of RNA-Seq and shotgun metagenomics are currently the most efficient and accurate technologies for large-scale profiling of rumen microbiomes (Cottier et al., 2018). Functional annotation of the rumen microbial genome is also important to evaluate the activity and function of the metagenome. However, microbial genomes are highly fragmented and currently the majority of DNA in metagenomes is very difficult to annotate. Some useful tools for functional annotation include enrichment analysis by annotation of metagenomic sequences to Kyoto Encyclopedia of Genes and Genomes pathways and Gene Ontology terms (Kanehisa et al., 2019). Ongoing research has shown continuous advancements in metagenomic sequencing technology allowing lower costs and higher throughput analysis, which will lead to improved characterization and identification of the microbial metagenome.

### Metagenomic profiling associated with methane emissions in ruminants

Metagenomic profiling has been applied to microbiome analysis in ruminant research, such as the characterization of the rumen microbial metagenome and its association with performance traits (Carberry et al., 2014a, b; Kamke et al., 2016; Roehe et al., 2016; Wang et al., 2017; Hess et al., 2020). A sophisticated network of symbiotic relationships exists between the (rumen) microbiota and the host, which are essential for metabolic maintenance, immune function, and overall production efficiency of the host (Hobson, 1997). In addition, many studies have investigated host–microbial interactions and their association with economically and environmentally important traits, including CH_4_ and FE in dairy cattle (Wang et al., 2017; Zhou et al., 2018), beef cattle (Zhou et al., 2009, 2011; Carberry et al., 2014a, b; Wallace et al., 2015; Roehe et al., 2016; Tapio et al., 2017), and sheep (Shi et al., 2014; Kamke et al., 2016; Rowe et al., 2019; Hess et al., 2020). For instance, Wang et al. (2017) identified rumen microbial genes encoding enzymes involved in methanogenesis pathways in dairy cattle. Higher *archaea* abundance and lower *Proteobacteria,* specifically *Succinivibrionaceae,* were shown to inhabit the rumen of higher CH_4_ emitting animals, suggesting that differences in microbial abundance may result in fluctuation of acetate and hydrogen production, potentially influencing methanogenesis and partially explaining the variation of CH_4_ in ruminants (Wallace et al., 2017). Similarly, Roehe et al. (2016) revealed that genes associated with methanogenesis’ pathways were divergent across CH_4_ cattle groups and 20 microbial genes were significantly associated with CH_4_. In sheep, a study of the rumen metagenome has enabled the identification of methanogenic pathways associated with CH_4_ phenotypes (Shi et al., 2014). In-depth metagenomic sequencing of archaeal species demonstrated a similar abundance of methanogens in high- and low- CH_4_ yielding sheep (Shi et al., 2014). Additionally, the integration of bacterial metagenome and metatranscriptome (bacterial gene expression) has enabled the identification of a high *Sharpea*-enriched bacterial community associated with low-CH_4_ yielding sheep (Kamke et al., 2016), suggesting that a lower hydrogen and CH_4_ producing methanogenic community inhabits the rumen of lower CH_4_ producing sheep. Lastly, Hess et al. (2020) developed a low-cost high-throughput approach to capture the diversity of the rumen microbiome between phenotypically extreme sheep for CH_4_. This approach is promising for obtaining metagenomic profiles and is comparable to that of bacterial 16S rRNA gene sequencing. Combining both metagenomic information and genomic information on large numbers of animals will allow predictions to be made for traits such as CH_4_ (Hess et al., 2020).

Production traits such as FE have also shown associations with CH_4_ when evaluating rumen microbial and metabolic pathway enrichment, supporting the influence of energy partitioning on CH_4_ and the importance of evaluating CH_4_ with correlated production traits (Shabat et al., 2016). Studies have further used metagenomic profiling, such as metataxonomy, to characterize rumen microbial profiles across divergent feed efficient cattle, and for example, revealed bacterial OTUs from specific bacterial families were present across divergent feed efficient beef heifers and steers (Paz et al., 2018). However, few studies have associated these production traits with CH_4_ traits using metagenomic technologies (Shabat et al., 2016).

The relationship between CH_4_ and FE is of great relevance when proposing alternatives to breed for lower CH_4_-emitting animals. Assessment of the rumen microbiome by evaluating the microbial ecology at the species, strain, or genotype level may assist in determining strategies for mitigating CH_4_ (Myer et al., 2015). It has been shown that more feed efficient cattle have lower methanogenic species variation and community diversity compared to inefficient cattle (Zhou et al., 2009, 2011). Furthermore, there is evidence that differing FE groups have similar metabolic pathways and host–microbial interactions that affect the methanogenic metagenome, which may influence CH_4_ (Guan et al., 2008; Zhou et al., 2009, 2011). However, other studies evaluating total methanogen abundance revealed no differences when comparing high and low FE groups (Zhou et al., 2009, 2011; Carberry et al., 2014a). Metagenomic analysis of rumen content can be used to identify microbial genes that are associated with CH_4_ and FE; thus, rumen microbial gene abundance may be used as a predictor of these traits (Roehe et al., 2016). In addition to rumen content and fluid sampling, the use of buccal swabs for metagenomic analysis as a noninvasive collection method and indirect way to assess the rumen metagenome has been studied, which revealed no significant differences in the microbial community across buccal or rumen sampling methods in sheep (Kittelmann et al., 2015). Further studies on the metagenomic analysis of biological samples such as saliva or buccal swabs, and associations with rumen metagenome, may lead to an indirect assessment of the rumen metagenome, providing further insight into the biology underlying CH_4_ in ruminants.

Metagenomic profiling of the rumen microbiome in different species and breeds shows promise of advancing our understanding of the diversity, function, and evolution of the microbial metagenome and its association with CH_4_. Metagenomic profiling has the potential to provide further insight into the associations between the host–microbiome interactions and host production efficiency (Delgado et al., 2019; Li et al., 2019), overall health (Malmuthuge and Guan, 2016), and nutrient metabolism (Wang et al., 2019). Further studies are necessary to determine links between host and rumen microbial metagenomic abundance, taxonomy, and function which can be used to predict important host phenotypes and performance. Metagenomic studies of the rumen microbiome will advance our understanding of rumen microbial diversity and community structure, and potential indications on microbial interactions and metabolism that may influence the regulation of CH_4_ in ruminants.

## Metabolomics

### Platforms and technologies available for metabolomics

Metabolomicanalysis has more recently become a major focus for “-omics” technologies for robust phenotyping in humans, crops, and model organisms, and its use and application in ruminant research has become more evident (Goldansaz et al., 2017; Huws et al., 2018). Metabolomics evaluates large quantities of metabolites, which enables the identification of physiological mechanisms and interactions between genes and the environment and has been applied to evaluate metabolite variations in milk, plasma, serum, urine, and ruminal fluid (Sun et al., 2015; Fontanesi, 2016; Goldansaz et al., 2017). These samples are commonly collected and assessed to provide insight on animal energy metabolism, by focusing on residual biomarkers. Comparing metabolites of individuals from divergent phenotype groups enables a better understanding of the biological processes that vary between individuals based on their genetic make-up, which could lead to differences in the phenotype observed.

There are numerous analytical instruments used to provide information about livestock metabolomes. The three most common methods are summarized with major advantages and limitations in Table 2. The most used platform is nuclear magnetic resonance (**NMR**) due to its high reliability (Goldansaz et al., 2017; Bica et al., 2020). This technique considers the spin properties of the nucleus of atoms, qualifying molecules, and metabolites present in the samples to be identified (Moco et al., 2007). Nuclear magnetic resonance enables metabolite identification in a nondestructive nature with minimal need for sample preparation time (Dettmer et al., 2007). However, chemical shift signals may cause clustering, making it challenging to identify individual metabolites in complex mixtures (Dettmer et al., 2007). A common method used in animal research involves NMR fingerprinting, which is based on collecting NMR spectra of complex mixtures of bio-fluids and comparing samples to pinpoint differences, enabling the identification of important biomarkers associated with the phenotypes of interest (Ratcliffe and Hill, 2005; Kochhar et al., 2006). Nuclear magnetic resonance technology is one of the most used techniques, however, it is relatively insensitive, as only substances in macromolar to millimolar concentrations are quantified (Goldansaz et al., 2017).

**Table 2.** Methods used and/or currently used to measure metabolites, and significant benefits and limitations for metabolomic analysis

Technique	Benefit	Limitation
Mass Spectrometry	Provides high selectivity and sensitivity for metabolite identification	Preparation step required can damage metabolites
Higher Performance Liquid Phase Chromatography	Allows the identification of compounds in a multi- component mixture	Costly process to run
Nuclear Magnetic Resonance	Nondestructive nature, minimal sample prep required	Individual metabolite identification in complex mixtures is challenging due to clustering

Mass spectrometry (**MS**) is another technique used in metabolomic studies, and unlike NMR, it can detect metabolites at nanomolar to picomolar concentrations (Goldansaz et al., 2017). Mass spectrometry provides high selectivity and sensitivity with the potential to identify metabolites, however, it requires a preparation step that can degrade metabolites from the sample and therefore they will be undetected (Dettmer et al., 2007). For this reason, to study the metabolome fully and comprehensively, liquid chromatography-MS (**LC-MS**) and gas chromatography-MS (**GC-MS**) have been combined with MS. Gas chromatography-MS is less sensitive than LC-MS, but it is generally more robust and reproducible (Goldansaz et al., 2017). Both LC-MS and GC-MS can detect a wide variety of compounds in biological samples. Liquid chromatography-MS utilizes soft ionization sources and atmospheric pressure chemical ionization. This method is very powerful as it has extremely high sensitivity, can reduce ion suppression, and is commonly used for the analysis of complex water samples and metabolomes (Dettmer et al., 2007; Ammann and Suter, 2016). In comparison, GC-MS uses hard-ionization methods and electron impact ionization and can analyze volatile and non-volatile compounds (Moco et al., 2007; Lei et al., 2011). As of 2017, the LC-MS technique has been used in approximately 25% of livestock metabolomic studies, while 15% of the livestock metabolomic studies used GC-MS, which is also common for other metabolomic disciplines (Goldansaz et al., 2017).

Ultimately, these approaches may yield useful information for the chemical quantification and identification of metabolites (Fontanesi, 2016). However, a potential challenge associated with these high-resolution metabolomic technologies is data management (Pieper et al., 2015). In the past, NMR was the preferred method of choice, but both GC-MS and LC-MS have become key analytical platforms for various types of metabolomic applications. This is due to the increasing reproducibility and higher sensitivity of these systems (Regal et al., 2011). In comparison, LC-MS appears to be the most versatile due to its ability to separate compounds with a broad range of polarity with minimal effort in sample preparation (Moco et al., 2007). However, to broaden the metabolite coverage, multiple metabolic platforms should be used.

### Metabolomic profiling associated with methane emissions in ruminants

The metabolomic profile of both human and livestock species display a continuous range of variation among individuals, and the amount of gut bacterial metabolites is presently undetermined (Dorrestein et al., 2014; Fontanesi, 2016). Uncovering these metabolomic profiles can lead to the understanding of biological processes essential to genetic and environmental differences among animals and could provide insight on innovative applications in animal breeding and management. In conjunction with genetic tools, metabolomics illustrates one of the biological levels to help understanding how the host genome influences an individual’s metabolism and consequently, the potential causes of phenotypic variability. A recent review highlighted the evident effect of host genetics on rumen microbial features; however, the extent to which these interactions are causal is not known (Newbold and Ramos-Morales, 2020). The use of metabolome data to indirectly assess CH_4_ offers the ability to collect data in a noninvasive and indirect collection of readily available samples such as milk. For instance, Antunes-Fernandes et al. (2016) investigated the milk metabolome of Holstein-Friesian cattle to identify the biological pathways associated with CH_4_ and reported four volatile (1-heptanol-decanol, 3-nonanone, ethanol, and tetrahydrofuran) and six nonvolatile metabolites (acetoacetate, creatinine, ethanol, formate, methylmalonate, and N-acetylsugar A) positively related with CH_4_ intensity, whereas, uridine diphosphate-hexose B, and citrate were negatively related with CH_4_ intensity (Antunes-Fernandes et al., 2016). This study supports the hypothesis that the milk metabolome is a valuable source to understand the biological metabolism of dairy cows in relation to CH_4_. Another group of researchers, Castro-Montoya et al. (2016), investigated the relationship between milk fatty acids (**MFA**) and CH_4_ in dairy cows. They found that the use of different methods for MFA collection and analysis between studies results in difficulty to identify a clear pattern on the association between MFA and CH_4_ (Castro-Montoya et al., 2016). Despite these findings, this is a promising area of research, and thus, there is potential to identify MFA biomarkers associated with CH_4_.

Further research in metabolomic profiling in ruminants is necessary to improve the current understanding of biological processes and biomarkers that play a role in the regulation of desirable traits (van Gastelen et al. 2018; Newbold and Ramos-Morales, 2020; Sun et al., 2020; Xue et al., 2020). Unfortunately, a large gap in research exists on studies assessing the metabolome and its association with CH_4_, and more specifically, on the understanding of the rumen microbial metabolome (Newbold and Ramos-Morales, 2020). The current understanding of the functional context of rumen microbiome metabolome is limited to our knowledge of defined and annotated rumen metabolites (Newbold and Ramos-Morales, 2020). However, as biomarkers have been insightful into higher milk yields and milk protein quality, there is a great opportunity for the investigation of the microbiome and its association with CH_4_ using metabolomics (Sun et al., 2015; Sun et al., 2020; Xue et al., 2020).

## Integration of “-Omics” Data with Functional Analyses to Identify Potential Candidate Regions and Genes Regulating Methane Emissions

Integration of high-throughput “-omics” technologies such as metabolomics, metagenomics, and host genomics with functional analyses using a systems biology approach can provide important information on functional genes and their influence on the regulation of complex trait phenotypes including CH_4_ (Cánovas et al., 2014a, b; Cánovas, 2016; Fonseca et al., 2018). Additional to metabolomics and metagenomics, transcriptomics is an “-omics” technology that evaluates the transcriptome at a high-throughput level, which consists of the total expressed RNA in a cell or tissue at a specific time point, and allows for measurement of gene expression levels, detection of structural variants including single-nucleotide polymorphisms (**SNP)**, insertions and deletions, and the identification of differentially expressed splice variants in the entire transcriptome (Wang et al., 2009). Such knowledge about genetic variation and gene expression can provide information on potential candidate regions and genes regulating CH_4_. A widely used tool for transcriptome and functional analysis includes RNA-Seq, which as previously mentioned, is used for transcriptome profiling to measure gene expression and genome structure through the identification of SNPs and other structural variants (Cánovas et al., 2010, 2013; Cardoso et al., 2018; Lam et al., 2020, 2021; Suravajhala et al., 2016). RNA-Seq quantifies all present genes and is more cost-effective, robust, accurate, and high-throughput compared to the traditional method of microarray analysis (Cánovas et al., 2010; Wickramasinghe et al., 2014). Metatranscriptomics uses RNA-Seq technology to evaluate community-wide function and activity of host tissue transcriptome (i.e., muscle, liver, fat) as well as the transcriptome of rumen microbes (i.e., rumen tissue, contents, or fluid), which can be coupled with other “-omics” technologies to integrate multiple levels of information using a systems biology approach (Kamke et al., 2016; Li et al., 2018; Huws et al., 2018). The integration of transcriptome and metatranscriptome data of the host and rumen microbe transcriptome, respectively, can provide information on similarities in the diversity, abundance, and differential gene expression in microbial communities and host genetics which may be associated with CH_4_ (Huws et al., 2018).

Following RNA-Seq analysis, several functional systems biology technologies can be applied from RNA-Seq data including, pathway analysis and Weighted Gene Co-Expression Network Analysis (**WGCNA**) which is most commonly performed on tissue RNA-Seq and provides information on networks and clusters of highly correlated genes to be linked to cattle FE (Kong et al., 2016). Few studies have used WGCNA network analysis in ruminant research due to the requirement of a large population size with RNA-Seq data. A recent study by Kong et al. (2016) on beef cattle divergent for FE, performed WGCNA and revealed three co-expressed gene clusters significantly correlated with RFI. Sun et al. (2019) also performed WGCNA to relate models of highly correlated genes to four FE-related traits. The authors concluded that combining confounding factors (breed types, start ages, body weight, tissues) with the weighted gene module–trait relationship contributes to a high coverage and powerful analysis of transcriptome interpretation (Sun et al., 2019). Other functional technologies include the concept of Genetical Genomics, which arose with the development of ruminant genome sequences, and investigates the gene expression of the entire transcriptome by integrating both structural and functional genomics data through a combination of gene expression and genotypic data (Cánovas, 2016; Cánovas et al., 2017). The use of the latter integrative analyses may provide further functional information on regulatory genes and gene networks associated with important traits such as CH_4_.

Combining and interpreting multiple levels of datasets resulted in the systems biology approach, allowing for a better biological understanding of phenotypes (Cánovas, 2016; Fonseca et al., 2018), as it allows for the study of interactions between components of biological systems and how the interactions influence the function and behavior of those systems (Cánovas et al., 2014a, b; Dias et al., 2017; Te Pas et al., 2017; Fonseca et al., 2018). Systems biology is especially useful to elucidate complex traits that are regulated by multiple physiological processes. Integration of different “-omics” datasets, such as metagenomics, metabolomics, transcriptomics, metatranscriptomics, and proteomics with systems biology tools such as network analyses and multilevel data integration can provide a more complete understanding of observed phenotypes in ruminant animals (Huws et al., 2018; Sun et al., 2020). Due to vast improvements in sequencing technologies, the efficiency and costs to obtain different levels of information at the “-omics” level have improved substantially (Suravajhala et al., 2016).

Genomic information from integrative “-omics” data and systems biology analysis on desirable traits can then be incorporated into breeding strategies to improve production efficiency (Cánovas, 2016; Suravajhala et al., 2016; Fleming et al., 2018). Information from integrated “omics” data and systems biology can then be disseminated and applied to industry to reveal more biological and genetic information to improve the understanding of complex and difficult to measure performance traits. This will result in increased accuracy of selection, a reduction in the generation interval, and an increased rate of genetic improvement of traits that are difficult to measure (Cánovas, 2016; Suravajhala et al., 2016). Overall, this may lead to improved competitiveness of the ruminant production industry through the improvement of economically and environmentally important traits such as CH_4_, using integrated “-omics” technologies and a systems biology approach.

Integrative approaches are currently being applied to uncover information associating different levels of omics, and further investigation of multi-level analysis is needed, such as integrating the genome, transcriptome, and metabolome. As the genome and transcriptome directly affect metabolite levels which result in changes to metabolite profiles; this highlights the importance of evaluating these levels as a system since specific metabolites are greatly affected by specific genetic features, and approaches are emerging to connect genomics with metabolomics. Integrative analyses include metabolite genome-wide association study (**mGWAS**), which uses GWAS to identify QTLs and significant SNPs associated with a metabolite. This allows identification of SNP-Metabolite trait associations or mQTL (Kadarmideen, 2016). In addition, machine learning algorithms have been used for clustering analysis. This includes multivariate analyses such as Random Forest analysis. Random forest is a supervised machine learning algorithm that operates with decision trees to classify specific features together (i.e., clustering multiple metabolites based on commonalities). Melzer et al. (2013) demonstrated the use of Random Forest and Partial Least Squares (**PLS**) to determine correlations between milk traits and metabolite profiles. To further integrate this approach with other -omics information, genetic correlations of metabolite profiles with traits such as CH_4_ emissions can be done.

To integrate rumen microbiome features, other machine learning approaches have been used in studies aiming to identify relationships between the microbiome and host phenotypes, including penalized regression, support vector machine, Random Forest, and artificial neural networks (Namkung, 2020). Although these analyses are already applied in human studies, there is a lack of studies using these approaches to firstly integrate -omics levels in livestock research, and secondly, to use the latter to better understand CH_4_ emissions at a system level.

## Conclusions

Due to the negative environmental impact of global warming, the demand to mitigate CH_4_ in ruminants is increasing. The development of cost-effective ways to mitigate CH_4_ has become a key priority worldwide, and this may be achieved by improving breeding strategies for more economically and environmentally efficient animals. Using previously collected data and existing resources in combination with high-throughput “-omics” technologies will advance our knowledge of the functional understanding of the metabolome and metagenome of ruminants. Increased integration of metabolomics and metagenomic information with other high-throughput “-omics” technologies and systems biology analyses are needed to provide a deeper understanding of the genes and metabolic pathways that influence the regulation and variation of CH_4_ production in ruminant animals.

## Abbreviations

FE: feed efficiency
GC-MS: gas chromatography-mass spectrometry
GHG: greenhouse gas
GWAS: genome-wide association study
ITS: internal transcribed spacer
LC-MS: liquid chromatography-mass spectrometry
LMBD: livestock metabolome database
MAGs: metagenomic-assembled genomes
MFA: milk fatty acid
mGWAS: metabolite genome-wide association study
MIR: mid-infrared
MS: mass spectrometry
NGS: next-generation sequencing
NMR: nuclear magnetic resonance
OTU: operational taxonomic unit
PCR: polymerase chain reaction
PEX2: *Peroxisomal Biogenesis Factor 2*
PME: predicted methane emission
qPCR: quantitative polymerase chain reaction
RFI: residual feed intake
RNA-Seq: RNA sequencing
SF6: sulfa hexafluoride
SNP: single-nucleotide polymorphism
TSPAN14: *Tetraspanin 14*
WGCNA: weighted gene co-expression network analysis
